# Rapid detection and simultaneous identification of the *Mycoplasma* and *Ureaplasma* species by real-time PCR and melt curve analysis among fertile and infertile females

**DOI:** 10.22038/IJBMS.2023.66170.14545

**Published:** 2023

**Authors:** Zeinab Fathizadeh, Hatave Ghasemi Tehrani, Mohammad Kazemi, Vajihe Karbasizade

**Affiliations:** 1 Department of Microbiology, School of Medicine, Isfahan University of Medical Sciences, Isfahan, Iran; 2 Department of Obstetrics and Gynecology, Isfahan University of Medical Sciences, Isfahan, Iran; 3 Department of Genetics and Molecular Biology, Isfahan University of Medical Sciences, Isfahan, Iran

**Keywords:** Infertility, Mycoplasma, Real-time PCR, Reproductive tract – infections, Ureaplasma

## Abstract

**Objective(s):**

*Mycoplasma* and *Ureaplasma* species threaten reproductive health and fertility worldwide. Due to the lack of sensitive, accurate, and affordable diagnostic tools, the simultaneous contributions of these agents in infertility have been overlooked. This study aims to detect and identify *Mycoplasma* and *Ureaplasma* species in the genital tracts of fertile and infertile females simultaneously.

**Materials and Methods:**

In a case-control study, cervicovaginal clinical samples were collected from patients referred to two teaching hospitals in Isfahan from July 2019 to February 2019. The initial screening was by using Real-time PCR and designed primer to evaluate the presence of *Mycoplasma *and *Ureaplasma* species including fertile and infertile women. The bacteria species were then detected and differentiated by using the melt curve and sequenced to confirm and identify. Finally, the standard curve was used to measure and compare the copy number of each species in each group. The isolates also were detected in clinical samples using the commercial PCR method.

**Results:**

The frequencies of *Mycoplasma genitalium* and *Mycoplasma* *hominis* were (0.0, 10.0%) in the fertile group and (4.3%, 34.3%) in the infertile group, respectively. *Ureaplasma* *parvum* and *Ureaplasma urealyticum* species in the fertile group (7.1%, 5.7%) and in the infertile group (32.9%, 24.3) were determined, respectively. The comparison of the results obtained from PCR and Real-time PCR showed that the recent technique has the ability to track 101–103 copy numbers.

**Conclusion:**

The present method allows differential diagnosis and quantification of *Mycoplasma* and *Ureaplasma* species in a short time and simultaneously.

## Introduction

According to the definition by World Health Organization (WHO), infertility is a disease in the reproductive system characterized by the failure to achieve clinical pregnancy during regular and unprotected intercourse after 12 months or more. Social problems, psychological effects, and clinical depression are all consequences of infertility, which are substantially greater in less industrialized countries. According to recent research, women have a 50% part in infertility (1-3). Secondary infertility means infertility after one year of the last pregnancy, and it is the most common type of female infertility worldwide (2, 4).

There are many causes of infertility, and infectious diseases are important agents of infertility (3). Infectious agents include *Neisseria gonorrhoeae*, *Chlamydia trachomatis*, *Treponema pallidum*, and urogenital *Mycoplasma*, which are sexually transmitted pathogens that threaten reproductive health worldwide (3, 5).

In recent years, *Mycoplasma genitalium*, *Mycoplasma hominis*, *Ureaplasma parvum*, and *Ureaplasma urealyticum* species have become increasingly clinically important. *Mycoplasma* species can cause infertility in women by various mechanisms, including non-gonococcal urethritis (NGU), endometritis, vaginosis, cervicitis, and pelvic inflammatory disease (PID) (6). Because of the slow growth of these bacteria, as well as lack of sensitive and simple diagnostic tools, their involvement in infertility has been overlooked (7). Using molecular methods to detect these isolates can be a more suitable alternative to non-sensitive serological and time-consuming methods, such as culture (8). TaqMan probe Real-time polymerase chain reaction is a sensitive method to detect *Mycoplasmas* and *Ureaplasmas*, but these methods are not only costly but have not detected these species simultaneously yet. Regarding the important roles of these organisms in infertility and developing sexually transmitted diseases, improving and changing sensitive molecular methods are necessary.

Numerous studies were conducted on the frequency of some species of *mycoplasma* and *ureaplasma* in fertile and infertile women separately in Iran and other regions of the world, and they have reported different results (9, 10). Rekha *et al*. found a link between* Mycoplasma* species and infertility, however, another study found no evidence of a link between the general distribution of these bacterial infections and infertility (10, 11). This disagreement goes back to the method of detecting and tracing these species and the geographical region. Considering the lack of information about the simultaneous frequencies of these microorganisms in fertile and infertile women in this geographical region, the present study aimed to determine the frequencies of *Mycoplasma* and *Ureaplasma* species in the genital tracts of the two groups who visited two teaching hospitals in Isfahan using the Real-time PCR technique and melt curve analysis.

## Materials and Methods

***Clinical specimens***

In the present case-control analytical study, the cervicovaginal swab specimens were taken from 140 patients who visited Shahid Beheshti and Al-Zahrai teaching hospitals in Isfahan during a period of 8 months. The inclusion criteria were: not taking antibiotics (at least 10 days before sampling) and immunosuppressive drugs, full patient satisfaction, married women between 15 and 42 years of age, not having heart disease, and hard genital surgeries. Exclusion criteria included: lack of proper sampling, inappropriate mental conditions, poor health of the reproductive system, hormonal problems, and history of hereditary infertility.

 The specimens were placed into microtubes containing 500 μl of sterile PBS and were transferred to the laboratory. After the centrifugation, they were stored at -70 °C until DNA extraction.

***Genomic DNA extraction***

Genomic DNA extraction from swab specimens was performed using the Favorgen Tissue Genomic DNA extraction kit (Favorgen, Taiwan) based on the manufacturer’s instructions. The quantity and quality of DNA extracted were examined by Nanodrop (Nanodrop 2000, Thermo Fisher Scientific, USA) at 260 and 280 nm and on the agarose gel. 

***In-house PCR assay***

Regarding the presence of some bacteria that were the normal flora of the urogenital tract, the 16S rRNA gene of the bacteria in the extracted DNA was tested for PCR inhibitors. DG74 (5’-AGGAGGTGATCCAACCGCA-3’) and RW01 (5’-AACTGGAGGAAGGTGGGGAT-3’) primers were used to amplify a 370bp fragment (Greisen *et al*., 1993). The specimens with negative PCR of 16S rRNA gene were removed, and positive samples were then examined for *M*. *genitalium*, *M. hominis*, *U.parvum*, and *U. urealyticum*.

***Real-time PCR assay for screening samples***

For the initial screening of cervicovaginal specimens, a pair of primers were designed for amplification of the 16S rRNA gene by AlleleID7.6 (Premier Biosoft) software and synthesized by Metabion Company (Switzerland) (Table 1). 

Real-time PCR was performed using StepOne Plus™ (Applied Biosystems) in a final volume of 10 µl. For each reaction, 5 μl of BioFactTM 2X Real-time PCR Master mix kit (BIOFACT, Korea), 0.5 μl of each primer (10 pmol/μl), 1 μl of DNA Template, and 3.5 μl of nuclease-free water were added. The thermal cycle included initial denaturation at 95 °C for 15 min, then 40 cycles at 95 °C for 20 sec, 60 °C for 30 sec, and 72 °C for 30 sec. The melt curve temperature program was performed from 60 °C to 95 °C with increasing temperature by 0.3 °C in each step. DNA of standard strains of *M. genitalium* (ATCC: 33530), *M. hominis* (ATCC: 15056), *U. parvum* (ATCC 27815T), and *U. urealyticum* (ATCC: 29557) was extracted as the positive control, and sterile distilled water was the negative control. For qualitative assessment, a Real-time PCR product was loaded and electrophoresed on a 1.5 percent agarose gel. Finally, the results were analyzed. The specimens were divided into groups based on the difference in Tm value and Real-time PCR product length

***PCR and DNA sequencing***

Several specimens were selected for confirmation and identification from the clinical specimens screened using the Real-time PCR technique. A forward primer, GPO-1 (5_-ACTCCTACGGGAGGCAGCAGTA-3_), and a reverse

primer, MGSO (5_-TGCACCATCTGTCACTCTG TTAACCTC-3_), were used to amplify an approximately 700-bp length (703 to 713 bp) of the 16SrRNA gene (12). Conventional PCR was performed using a thermal cycler (Eppendorf Co, Germany) apparatus for the specimens. PCR was performed in a final volume of 25 µl. For each reaction, 12.5 μl of BioFact 2x PCR Master mix kit (BIOFACT, Korea), 1 μl of each primer (10 pmol/μl), 1 μl of template DNA, and 9.5 μl of nuclease-free water were added to each mixture. PCR program included initial denaturation at 94 °C for 10 min, and then 40 cycles of amplification as follows: Denaturation at 94 °C for 30 sec, annealing at 64 °C for 30 sec, extension at 72 °C for 60 sec, and final extension at 72 °C for 5 min. Finally, the PCR product was sequenced.

***Quantification of Urogenital Mycoplasmas by real-time PCR assay***

The purified PCR products were ligated into the pGEM-T Easy cloning vector (Promega) overnight at 4 °C in a 10 µl reaction mix. The ligated product was transformed in competent *Escherichia coli* DH5α cells to obtain plasmid clones containing inserted standard target DNA.

 The transformants obtained on LB agar plates with ampicillin were picked up and then inoculated in LB broth containing ampicillin and incubated in a shaking incubator at 37 °C for 16 hr.

Plasmid was extracted using FavorPrep Plasmid Extraction Mini Kit (Favorgen. Taiwan). The cloned copies were also confirmed for the presence of standard target DNA by PCR using primers specific to the target gene. The concentration of cloned plasmids was determined using Nanodrop 2000 (Thermo Scientific, USA) and the copy number of target DNA was calculated. To quantify each organism in the specimen, a series of consecutive 10-fold dilutions consisting of 5 standards (× to 1× copies per microliter) were prepared to draw the standard curve. To evaluate the standard curve, each standard sample was tested with three replications; finally, the number of copies of each target gene was determined in unknown specimens.

***PCR assay for screening samples***

Mycoplasma and Ureaplasma isolates were detected in clinical samples using the PCR method. The same primer pairs that were used in the Real-time PCR section were used in the conventional PCR to directly compare the two PCR methods. Reaction mixture volume was 25 µl and it contained 12.5 μl of BioFact 2x PCR Master mix kit (BIOFACT, Korea), 1 µl of each forward and reverse primers (10 pmol/μl), 1 μl of template DNA, and 9.5 μl of nuclease-free water. The PCR program consisted of initial denaturation at 95 °C for 10 min, followed by 40 cycles consisting of denaturation at 95 °C for 20 sec, annealing at 60 °C for 30 sec and extension at 72 °C for 30 sec, and a final extension at 72 °C for 5 min. Product and DNA marker were loaded and electrophoresed on a 1.5% agarose gel.

It is worth mentioning that due to the lack of copy numbers in the range of 101 to 102 in clinical samples, the desired serial dilutions were prepared using standard strains and used to evaluate the sensitivity of the PCR method.

***Statistical analysis***

The data were analyzed using SPSS 24. The frequency distribution tables and statistical graphs were used in the descriptive phase. The chi-square test, Fisher’s exact test, and logistic regression were utilized in the inferential phase.

## Results

***Real-time PCR assay***

The designed primer was first evaluated on standard strains, a part of the 16SrRNA gene of Mycoplasmas and Ureaplasmas was amplified at approximately 448 bp and 557 bp, respectively using the Real-time PCR and the designed primer. Melt curve analysis of each amplicon showed two distinct peaks with different Tm, each of which indicated a single species in that genus (Table 1).

***Screening clinical samples***

**Figure 1:**
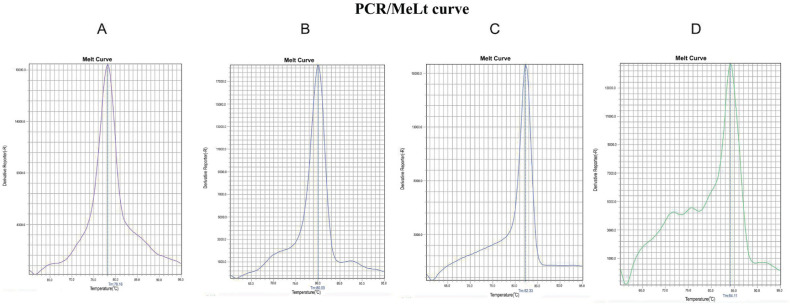
Comparison of melt curve diagrams of clinical samples and their DNA sequencing results; Graphs are related to clinical samples with positive Real-time PCR/Melt curves. Graph (A), *Mycoplasma genitalium* with Tm value of 78.10; graph (B), *Mycoplasma hominis* with Tm value of 80.0; graph (C), *Ureaplasma parvum* with Tm value of 82.30; graph (D),* Ureaplasma urealyticum* with Tm value of 84.10

**Figure 2:**
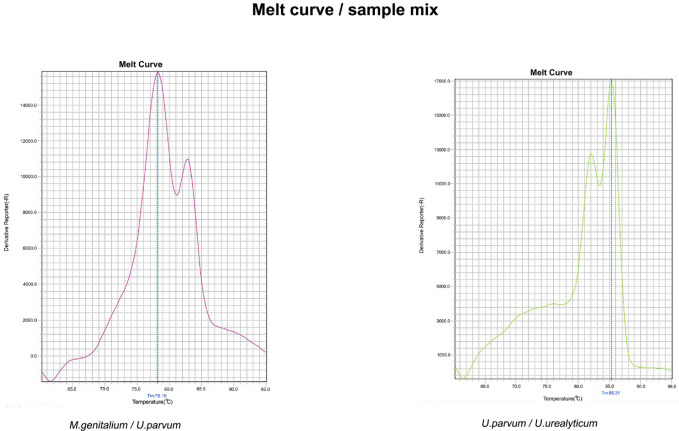
Using Real-time PCR technique and melt curve provided the quantitative and qualitative detection, as well as simultaneous differentiation of *Mycoplasma* and *Ureaplasma* species. Graph (A), *M. genitalium* and *U. parvum*; graph (B), *U. parvum* and *U. urealyticum*

Among 140 cervicovaginal samples, 83 samples were positive for the presence of *Mycoplasma* and *Ureaplasma* species, of which 67 specimens belonged to the infertile group, and 16 specimens belonged to the fertile group. The analysis of the melt curve indicated four different peaks belonging to *M. genitalium, M. hominis, U. parvum*, and *U.* *urealyticum *species (Figure 1). In the mixed samples that had both *Mycoplasma* and *Ureaplasma *species, the Real-Time PCR product showed different amplicons with an approximate size of 450 bp (exact size of 465 bp and 448 bp) and 550 bp (exact size of 557 bp and 558 bp). The amplicon whose size was in the range of 450 bp was related to* Mycoplasma* species, which depending on the species had a melting profile of Tm = 80.5 ± 0.5 and Tm = 78.5 ± 0.5. The amplicon that was in the range of 550 bp related to *Ureaplasma *species, which depending on the melting profile showed Tm = 82.5±0.5 and Tm = 84.5±0.5. In samples containing only *Mycoplasma* or *Ureaplasma* species, the Real-Time PCR product showed an amplicon, however, its melting profile was different depending on the presence of one or more species (Figure 2). Sequencing and aligning in the NCBI database using BLAST validated the identification of distinct *Mycoplasma* and *Ureaplasma* species.

**Figure 3:**
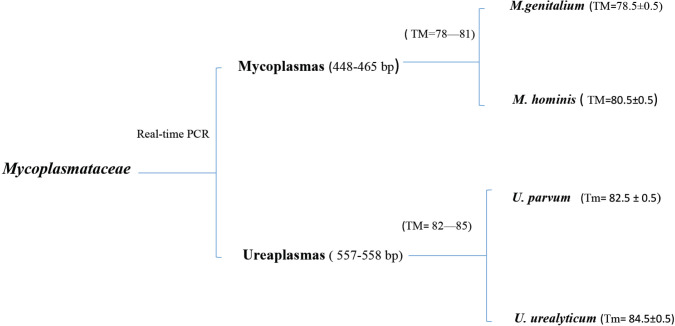
Urogenital *Mycoplasma* was differentiated at the genus and species levels using Real-time PCR product and melt curve analysis

Therefore, melting curve analysis of clinical samples showed four peaks with different Tm. After comparison with the melting curve of positive control strains and sequencing some of them, the samples were divided into four groups: A, B, C, and D (Figure 3). 

***Prevalence of Mycoplasma in fertile and infertile women***

The distribution of *Mycoplasmacae* species in two groups indicated that the frequencies of *Mycoplasma* and *Ureaplasma* species, as well as their simultaneous colonization, were significantly higher in the infertile group than in the fertile group. The difference was statistically significant for all species, except for *M. genitalium* (*P*<0.001, Table 2).

The copy number of *Mycoplasma* and *Ureaplasma* species in the swab samples of the two groups was calculated by standard curve analysis.

In the infertile group *M. hominis* load ranged from to copies/μl, *M. genitalium* ranged from to copies/μl, *U. parvum* ranged from to copies/μl, and *U.* *urealyticum* ranged from to copies/μl. However in the fertile group *M. hominis* load ranged from tocopies/μl, *M. genitalium* ranged from 0 copies, *U. parvum* ranged from to copies/μl, and *U. urealyticum* ranged from to copies/μl. The mean number of copies of *Mycoplasma *and *Ureaplasma* species in swab samples was higher in the infertile group than in the fertile group.

Demographic studies between the two groups indicated that the mean age of the infertile group was 32.3±5.81 and that of the fertile group was 32.8±6.54. The highest frequencies of *Mycoplasma* and *Ureaplasma* species were observed in the age group of 30–34 years, which was 35.7% in the infertile group and 27.1% in the fertile group. Individuals with a high school education had the greatest frequency of the species, which was 45.7 percent in the infertile group and 60.0 percent in the fertile group. There is no significant relationship between demographic indicators and the presence of genitourinary mycoplasmas (*P*>0.05).

Among 70 infertile women, 77.1% (n=54) had primary infertility and 22.9% (n=16) had secondary infertility. The duration of infertility varied from 1 to a maximum of 20 years among women with primary and secondary infertility. *U. parvum* and *M. hominis* were the most common organisms in primary and secondary infertility patients, respectively. However, the prevalence of *Mycoplasma* and *Ureaplasma* did not show any significant relationship with the type of infertility (*P*>0.05; Table 3).

***Standard***

**Figure 4:**
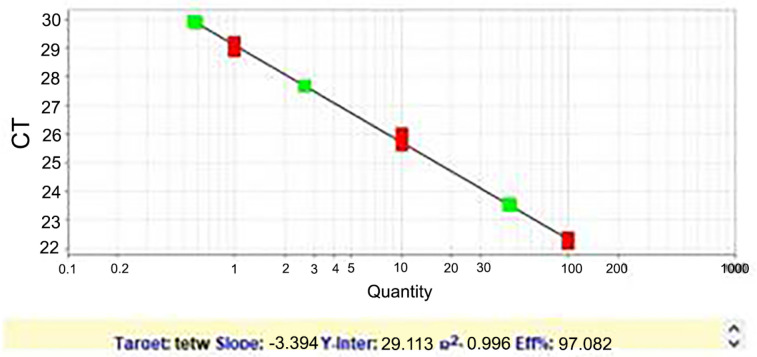
The standard curve of urogenital mycoplasmas, a series of consecutive 10-fold dilutions consisting of 5 standards

Figure 4 shows the standard curve for quantification and comparison of samples.

***Comparison of real-time PCR with conventional PCR method***

The comparison of the results obtained in 140 clinical specimens by Real-time PCR procedure with the conventional PCR method is shown in Table 4. The results of 70 infertile women were analyzed; 27 specimens (38.5%) were positive for *Mycoplasma* species and 40 specimens (51.7%) were positive for *Ureaplasma* species by both conventional PCR method and Real-time PCR. While in the fertile group, out of 70 specimens, 7 patients (10.0%) were positive for *Mycoplasma* species and 9 patients (12.8%) were positive for *Ureaplasma* species by Real-time PCR method, 4 patients (5.7%) were positive for *Mycoplasma* species and 6 patients (8.5%) were positive for *Ureaplasma* species only by conventional PCR. The copy numbers were in the range of to copies/μl for *Mycoplasma* species and to copies/μl for *Ureaplasma* species in positive samples in infertile women. In fertile women, *Mycoplasma* species were in the range of to copies/μl and for *Ureaplasma* species to copies/μl.

**Table 1:** Primers used in Real-time PCR assay for amplification and detection of *Mycoplasma* and *Ureaplasma*

Melting Temperature	Size of Product (bp)	Species	Genus	Sequence (5’–3’)	Name
78.5 ± 0.5 80.5 ± 0.5 82.5± 0.5 84.5 ± 0.5	465 448 557 558	***M. genitalium*** ***M. hominis*** ***U. parvum *** ***U. urealyticum***	Mycoplasmas Ureaplasmas	TGGRGTKAAGTCGTA CTGAGATGTTTCAMTTCACM	Myc F MycoR

**Table 2:** Frequency of urogenital *Mycoplasma* and *Ureaplasma* species in fertile and infertile women groups

Mycoplasma*s*	Infertile (n=70)		fertile (n=70)		*P*-vlaue*
	n	%	n	%	-
*M.* *hominis*	24	34.3	7	10.0	0.001
*M. genitalium *	3	4.3	0	0.0	0.245
*U.* *parvum*	23	32.9	5	7.1	<0.001
*U. urealyticum*	17	24.3	4	5.7	0.002
*mix*	17	24.3	2	2.9	<0.001

* Data were analyzed using Fisher's exact test and Chi-square

**Table 3:** Prevalence of *Mycoplasma* and *Ureaplasma* species in women with primary and secondary Infertility

Mycoplasma*s*	Infertility						*P*-value
	Primary (n=54)		Secondary (n=16)		Total (n=70)		
	n	%	n	%	n	%	
*M.* *hominis*	17	31.5	7	43.8	24	34.3	0.364
*M. genitalium*	1	1.9	2	12.5	3	4.3	0.129
*U.* *parvum*	20	37.0	3	18.8	23	32.9	0.171
*U. urealyticum*	13	24.1	4	25.0	17	24.3	0.940
mix	14	25.9	3	18.8	17	24.3	0.557

**Table 4:** Comparison of Real-time PCR with conventional PCR method for detecting *Mycoplasma* and *Ureaplasma* in cervicovaginal specimens from infertile and fertile women

**Subjects**		**No. (%) of specimens with a positive result**								
	**In** **fertile (n=70)**					**Fertile (n=70)**				
	**Convention PCR (%)**	** Real-time quantitative PCR (%) **		**Convention PCR (%)**			**Real-time quantitative PCR (%) **		
*** Mycoplasma***	**27 (38.5)**	** 27 (38.5)**			** 4 (5.7) **			**7 (10.0)**
***Ureaplasma***	** 40 ( 51.7)**	** 40 ( 51.7)**	**6 (8.5** **)**					**9 (12.8** **)**
**mix**	**17 (24.3)**	**17 (24.3)**	**2 (2.9)**					**2 (2.9)**

## Discussion

Identification of *Mycoplasma* and *Ureaplasma* species can lead to quick approaches to control their acute and chronic infections. Molecular methods take less time to identify *Mycoplasma* and *Ureaplasma* species of the genital tract than culture-based methods** (**11). The simultaneous detection and differentiation of *Mycoplasma* and *Ureaplasma* species are not possible with the help of conventional PCR without positive control, and it requires sequencing to detect some species (13). Real-time PCR can be used as a useful alternative to conventional molecular techniques for rapid and accurate detection of *Mycoplasma* and *Ureaplasma* species. We presented a method for simultaneous detection of *Mycoplasma* and *Ureaplasma* species based on differentiation of melting temperature from 16SrRNA gene amplicons in clinical samples using a Real-time PCR technique. By studying the melt curve, *Mycoplasma* and *Ureaplasma* species may be distinguished in less than 5 hr without the necessity for sequencing in a single test. High sensitivity, simultaneous identification of *Mycoplasma* and *Ureaplasma *species in clinical specimens, and a shorter reaction time are all benefits of this technology over conventional methods like PCR and culture.

There are disagreements or arguments about the exact relationship between *Mycoplasma* and *Ureaplasma* species with human infertility. Previous studies have shown that in vaginal dysbiosis, these microorganisms cause infections that have adverse effects on fertility; however, other studies have not shown the impact of these infections and their association with infertility. Species differentiation provides great value to prove their correlation and pathogenicity. In the present study, the frequencies of *U. urealyticum*, *U. Parvum*, and *M. hominis* species in the urogenital system of infertile women were significantly higher than in fertile women. The results were consistent with previous reports of a high presence of these bacterial species in infertile women (3, 9, 14). In other studies, the frequency of some of these species in fertile and infertile groups did not vary significantly (9). Differences in results may be in terms of differences in the socio-economic conditions of the population, sampling place and method, living standards, and most importantly, diagnostic methods. Among these three microorganisms, the frequency of *M. hominis* was higher in the two groups. Among these three mentioned organisms, the frequency of M. hominis was higher in the infertile group, while some other studies reported that the most common microorganism was *U. urealyticum*. The existing differences can be explained due to the high prevalence of vaginosis in the studied population. Studies have shown that *M. hominis* can be one of the important causes of bacterial vaginosis (15). 

Differentiation of *Ureaplasma* species can provide important information about the epidemiology of infections and their role in pathogenicity and infertility. *Ureaaplasma* *urealyticum* and *Ureaaplasma parvum* species are genetically closely related to each other, for this reason, limited studies using very sensitive and expensive methods such as real-time TaqMan PCR have separated these two species from each other (3-5). In the present study, with the help of a Real-time PCR procedure followed by melting curve analysis, using the green fluorescence dye SYBR Green, this microorganism was quantitatively detected and simultaneously differentiated. The results of this study showed that after *M. hominis, Ureaplasma parum* was the most common species. While some previous studies reported *U. urealyticum* as the most common organism, in which no distinction was made between *U. urealyticum* and *U. parvum* species, which were genetically closely related to each other (14, 16, 17). The differentiation between these two species requires the use of precise molecular techniques such as Real-time and sequencing, which have not been done in previous studies.

There was not any significant difference among the frequencies of *M. genitalium* in the two groups. The finding was consistent with the studies in Australia and Poland (16, 17) but inconsistent with studies by Sharma Rekha in India and Helle Friis Clausen in Denmark (11, 18)*. M. genitalium* is a neglected sexually transmitted pathogen among women, with a high prevalence among high-risk populations, including women with sexually transmitted infections and sex workers (19). Mono-partnering and restrictions on patients’ sexual relations can be reasons for the lower prevalence of this microorganism in the present study.

The simultaneous colonization of *Mycoplasma *and *Ureaplasma* species in the urogenital tract of infertile women was much greater than in fertile women, according to our findings. The high incidence of *mycoplasma* infections in the infertile group might be due to hormonal disorders in this group (9). Hormonal disorders can lead to lower immunity in infertile women, followed by higher colonization and bacterial survival in the vaginal epithelium (20).

The studies on these microorganisms show that the populations were socio-economically different (age and literacy) (21). The difference was also detected in the current research; hence, this problem is most likely the cause of conflicting data on the involvement of demographic factors in infertility.

Even though there was no significant difference between the fertile and infertile groups in terms of socio-economic indices (age, literacy), the highest rate of infection with mycoplasma and ureaplasma infections in the two groups belonged to the age group of 30–34 years with a high school diploma. The increase in mycoplasma infection among Iranian women in this age group was related to an increase in the average age of marriage and the fact that they had the most sexual activity (22). This study’s results were consistent with two other studies (23, 24) and different from the reports of Gupta *et al.* (25). 

Mycoplasma and Ureaplasma frequencies among women with primary and secondary infertility were not statistically significant because other bacterial agents, such as *Chlamydia trachomatis*, *Neisseria gonorrhea*, and *Trichomonas vaginalis*, which were involved in infertility (26), were not examined. Women with more sexual partners have an increased incidence of *Mycoplasma* and *Ureaplasma* strains in secondary infertility (26). Our findings indicated a higher incidence of these organisms in women with primary infertility than in women with secondary infertility. A reason for such difference was the mono-partnering of Iranian married women. Our results were consistent with the findings of another study (27).

Several studies have recently focused on bacterial load. Contini *et al*. showed a significant relationship between the number of *U. parvum* copies and histological chorioamnionitis (28). Huang suggests that carrying *U. parvum* at a high load may have significant consequences for the development and progression of cervicitis in women (29). Like some previous studies, the quantification of any species is important to study its pathogenicity. The high copy numbers of *Mycoplasma* and *Ureaplasma* species in the infertile group compared with the fertile group confirmed the importance of these organisms in infertility

In this study, the results obtained from PCR and Real-time PCR methods for the detection of *Mycoplasma* and *Ureaplasma* species were compared. In the two studied groups, samples with copy numbers of more than 10^3^ were positive with both methods. While samples with copy numbers in the range of 10^1^ to 10^3^ could only be traced using the Real-time PCR method, which shows higher sensitivity than PCR of the Real-time PCR technique These results were consistent with Xuan’s study (30). However, one limitation should be noted; in this study, the culture was not used as one of the diagnostic methods, which should be considered in future studies to determine the sensitivity and specificity of this method.

## Conclusion

To the best of our knowledge, this is the first study that has enabled the detection and simultaneous identification of the *Mycoplasma* and *Ureaplasma* species with the least cost and time using the Real-Time PCR technique and melt curve. Over the next few years, the results of previous and present studies are expected to provide a new perspective for the emergence of accurate and sensitive molecular methods for tracing *Mycoplasma *and *Ureaplasma* species. Our data showed a high prevalence of *M. hominis*, *U. urealyticum*, and *U. parvum* infections and low rate of *M. genitalium *in the infertile group compared with the fertile group. According to this study’s results, the diagnosis and control of these organisms among infertile people are important, and their detection can lead to measures to control infection, fertility, and infertility problems.

## Authors’ Contributions

All authors contributed to the study’s conception and design. VK, ZF, HGT, and MK performed material preparation, data collection, and analysis. ZF wrote the first draft of the manuscript, and all authors commented on previous versions of the manuscript. All authors read and approved the final manuscript.

## Funding

This study was supported by the Department of Microbiology, Faculty of Medicine, Isfahan University of Medical Sciences, Isfahan, Iran (Grant no. 399014).

## Ethical Approval

The study was approved by the Ethics Committee of Isfahan University of Medical Sciences, Iran (IR.MUI.MED.REC.1399.172/May 2, 2020). 

## Conflicts of Interest

There are no conflicts of interest.
